# Impact of Nutritional Interventions on Alzheimer’s Disease: A Systematic Review and Meta-Analysis

**DOI:** 10.7759/cureus.49467

**Published:** 2023-11-27

**Authors:** Rehana Basri, Mubarak Alruwaili, Raed AlRuwaili, Anas Mohammad Albarrak, Naif H Ali

**Affiliations:** 1 Department of Internal Medicine/Neurology, College of Medicine, Jouf University, Sakaka, SAU; 2 Department of Internal Medicine, College of Medicine, Prince Sattam Bin Abdulaziz University, Al-Kharj, SAU; 3 Department of Internal Medicine, Medical College, Najran University, Najran, SAU

**Keywords:** alzheimer’s disease, vitamins, nutritional profile, minerals, dietary habits, alzheimer's illness

## Abstract

The most prevalent type of dementia, especially in older persons, is Alzheimer's disease (AD), which has clinical signs of progressive cognitive decline and functional impairment. However, new research indicates that AD patients' dietary patterns and nutritional intake could hold the key to staving off some of the complications. Therefore, the primary aim of this investigation was to analyze various dietary patterns and the subsequent impact of the resulting nutritional intake on AD patients. Various online databases (PubMed, Scopus, Web of Science, and Google Scholar) were searched using appropriate keywords, reference searches, and citation searches. The databases were accessed using the search phrases "Alzheimer's disease," "dietary habits," "minerals," "nutritional profile," and "vitamins." Fifteen of the 21 investigations that we selected for our systematic review and subsequent meta-analysis revealed that micronutrient supplementation and some dietary patterns were helpful in alleviating a few of the symptoms of AD, especially with regard to the progression of dementia in the assessed patients. It was shown that dietary interventions and nutritional adjustments can considerably delay the onset of AD and the varying degrees of dementia that often accompany it. However, there were some areas of ambiguity in our findings because a few of the chosen studies did not document any noticeable improvements in the patient's conditions.

## Introduction and background

Alzheimer’s disease (AD), a neurological condition that primarily affects elderly people and is the leading cause of dementia and cognitive decline, affects about 50 million people worldwide [1]. In the past, high-income countries (West Europe and North America) had the greatest rates of AD prevalence; however, the incidence of dementia has recently begun to increase in low- and middle-income countries, with 63% of all dementia cases anticipated to occur in these countries by 2030 and 71% by 2050 [1].

Aging is a significant risk factor for AD, which is why it mostly affects those over 65 years, with AD that progresses as people age being referred to as sporadic AD [2]. However, the cause of this prevalence of AD in the elderly is still unknown [3]. For instance, studies have linked dietary protocols to AD progression [4-7]. Type 2 diabetes and obesity are other potential risk factors for AD [4-7]. The Mediterranean diet (MD) and ketogenic diets have been linked to healthy brain aging and a decreased risk of AD [4-7]. The fact that AD prevalence has increased in response to the epidemiological shift in developing countries, where the percentages of obese and diabetic people are rising, lends more credence to this theory [1]. Gut microbiota has also been shown to significantly affect AD pathogenesis [2,8-10]. Despite the fact that the pathogenesis and contributing factors for familial AD are yet unknown, genetic anomalies have been investigated and shown to promote neurotoxic accumulations in the brain [10]. Pathological extracellular neurofibrillary plaques and intracellular hyperphosphorylated tau (pTau) tangles are abundant in the cortical parenchyma of AD brains, especially in the temporal lobes [10]. The diagnosis of AD may also be supported by significant brain shrinkage. These visible symptoms have long been thought to be the root causes of AD pathology. In the neocortex, the buildup of tau neurofibrillary tangles and A plaques ultimately causes neurodegeneration [9,10]. The tau tangle and A plaque hypotheses have been considered to be the greatest illustrations of AD's pathogenesis. However, recent studies have linked AD to systemic dysfunction, which is at least partially mediated by gut microbiota and persistent, systemic, and brain inflammation. This idea of neuroinflammation includes the gut-brain axis, which links the actions of the gut microbiota to the well-being and performance of neurons [10].

The primary need for an investigation such as ours is to provide a comprehensive overview of the most current evidence in this area. Several studies have been done to address the impact of dietary interventions on cognitive decline and AD [11,12]; however, they have lacked in terms of assessing the impact of different dietary patterns and nutritional supplementation on AD in a direct manner. By focusing on recent studies, the review can provide an up-to-date assessment of the effectiveness of nutritional interventions for AD and provide directions for future research. Hence, by means of this systematic review and meta-analysis, we aimed to analyze the different types of dietary interventions and their nutritional profiles and ultimately assess their impact on the well-being of AD patients.

## Review

Materials and methods

Protocol Employed

**Figure 1:**
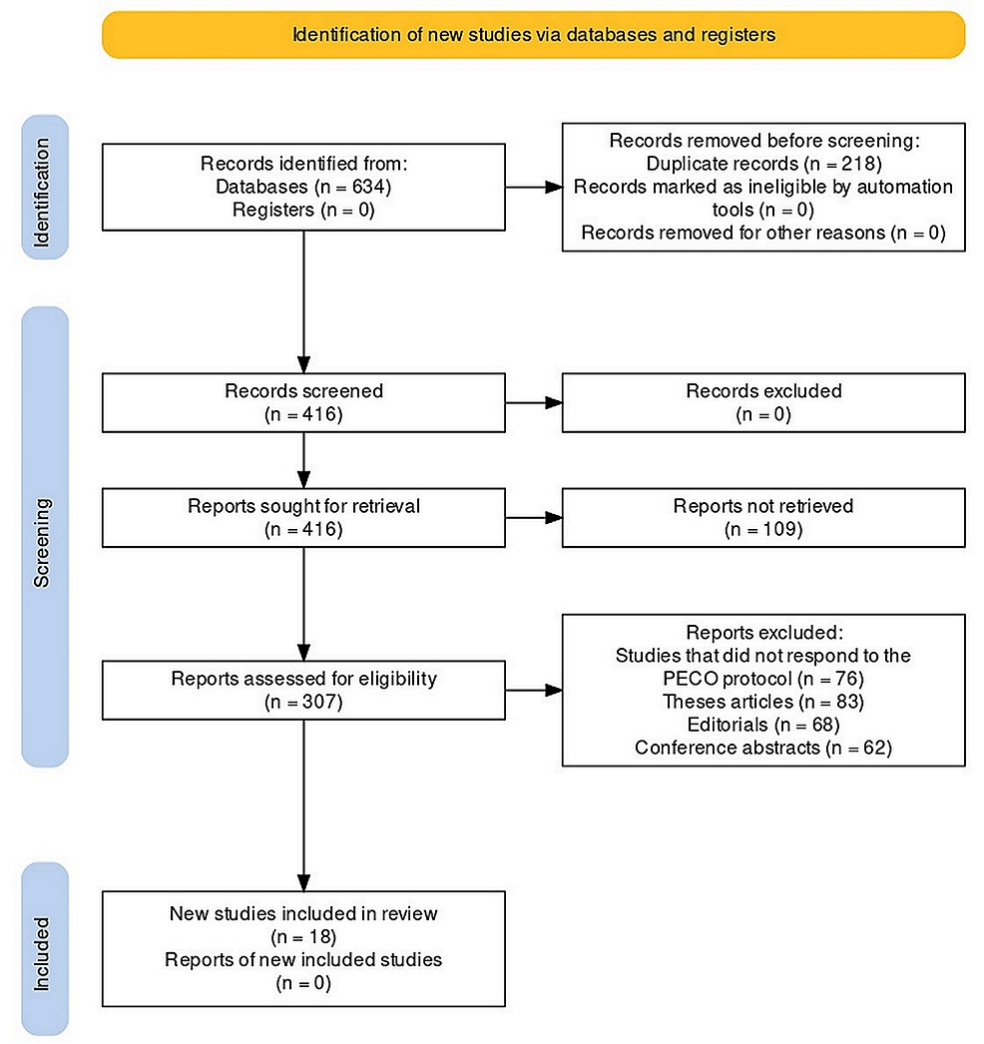
PRISMA flow chart PRISMA: Preferred Reporting Items for Systematic Reviews and Meta-Analyses.

This systematic review was performed as per the Preferred Reporting Items for Systematic Reviews and Meta-Analyses (PRISMA) protocol with the International Prospective Register of Systematic Reviews (PROSPERO) registration number CRD42023387059 [13]. The articles selected for the review using the protocol are depicted in Figure 1.

Review Objectives/Clinical Assessment Target(s)

The primary clinical objective of this systematic review was to analyze studies about how nutritional interventions and different diets, specifically diets like the ketogenic diet (KD) and Mediterranean diet (MD) or a combination of both, can affect people with AD. These studies were found in neurological literature.

Study Selection Criterion

The systematic review employed a specific inclusion criterion strategy to select relevant studies. The types of studies considered for inclusion encompassed clinical trials with appreciable sample sizes, systematic reviews that aligned with the review's objectives, as well as cohort studies, cross-sectional studies, and meta-analyses. The review specifically focused on adults who had a confirmed diagnosis of AD and studies that investigated nutritional interventions. These interventions could vary and include elements such as dietary patterns, vitamin and mineral supplementation, and changes to the nutritional profile. Furthermore, the review considered studies that examined cognitive function and brain volume related to AD, and only those published in the English language were included.

Conversely, there were certain types of studies and scenarios that were excluded from the review. Studies involving participants under the age of 18 years were excluded, as were studies that did not focus primarily on AD. In addition, studies that did not incorporate a nutritional intervention as part of the study's interventions were ruled out. The review omitted studies with a sample size of fewer than 10 participants per group. Other types of publications, such as conference abstracts, letters, editorials, and case reports, were also excluded. Finally, the review did not consider studies that had not undergone the peer-review process.

Search Strategy

The following search strategy was utilized for the systematic reviews.

PubMed: "Alzheimer's disease" AND "dietary patterns" AND "minerals" AND "nutritional profile" AND "vitamins." Limiters: humans, English language, peer-reviewed articles, and publication date range (last five years) utilizing Medical Subject Heading (MeSH) terms and Boolean operators.

Scopus: "Alzheimer's disease" AND "dietary patterns" AND "minerals" AND "nutritional profile" AND "vitamins." Limiters: humans, English language, peer-reviewed articles, and publication date range (last five years).

Web of Science: "Alzheimer's disease" AND "dietary patterns" AND "minerals" AND "nutritional profile" AND "vitamins." Limiters: humans, English language, peer-reviewed articles, and publication date range (last five years).

Google Scholar: "Alzheimer's disease" AND "dietary patterns" AND "minerals" AND "nutritional profile" AND "vitamins." Limiters: humans, English language, peer-reviewed articles, and publication date range (last five years).

Data Selection and Coding

Two reviewers extracted data from the selected articles using a standardized data extraction form separately. A variety of variable factors, including author, publication year, country, publication type, major findings, and conclusions, were included in the data that were extracted. The data extracted were then compared to ascertain consistency, with disagreements between the reviewers being resolved by a third independent reviewer wherever required. Then, in the final step, the synthesized data were assessed for quality using a specific validated tool (such as Assessment of Multiple Systematic Reviews (AMSTAR) for this systematic review).

Risk of Bias Assessment

**Figure 2:**
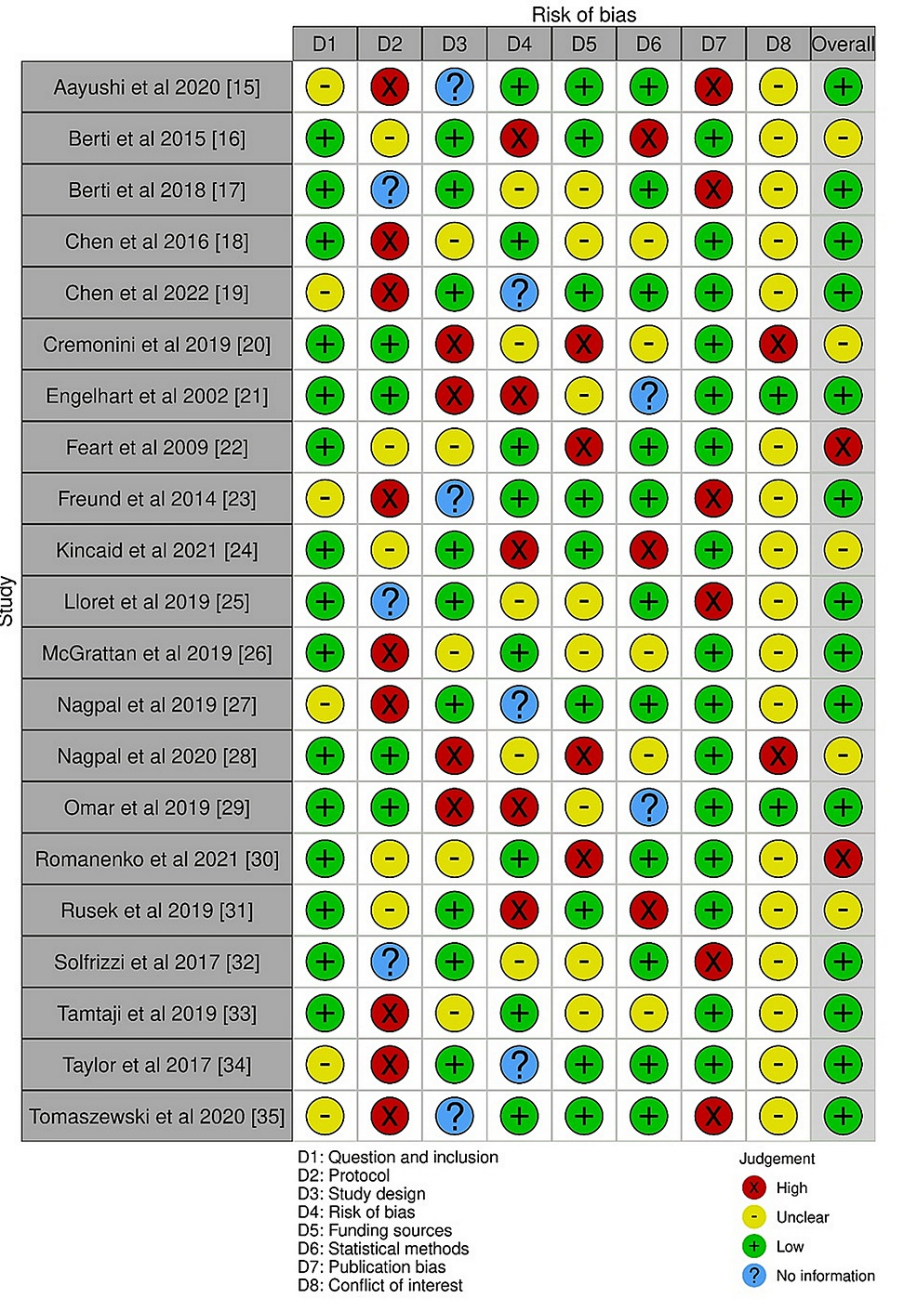
Risk of bias in the literature reviews and systematic reviews

The AMSTAR 2 tool [14] was used to evaluate the risk of bias in the literature reviews and systematic reviews that we chose for this study (Figure 2).

Statistical Analysis

Our study's meta-analysis produced forest plots that display the odds ratio for different study methodologies, when data on sample size, analyzed variables, and other investigation elements were carefully selected and subsequently entered into the RevMan 5 software (Cochrane Collaboration, London, UK) for meta-analysis.

Results

**Figure 3:**
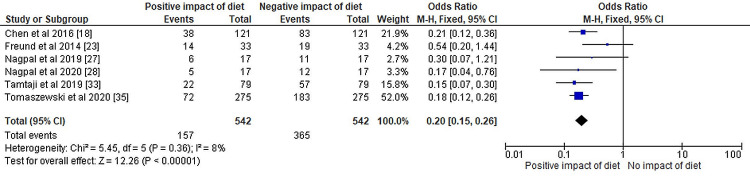
The odds ratio of selected randomized control trials represented on a forest plot after their meta-analysis where the positive impact of dietary interventions compared to the negative to negligible impact of dietary patterns was assessed

**Figure 4:**
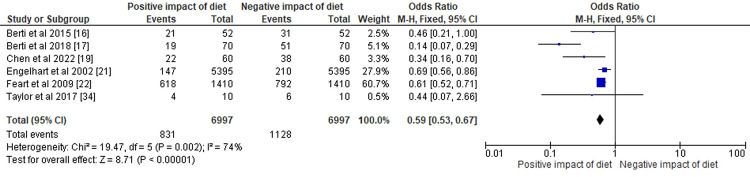
The odds ratio of selected cross-sectional, observational, and cohort-based studies represented on a forest plot after their meta-analysis where the positive impact of dietary interventions compared to the negative to negligible impact of dietary patterns was assessed

**Figure 5:**
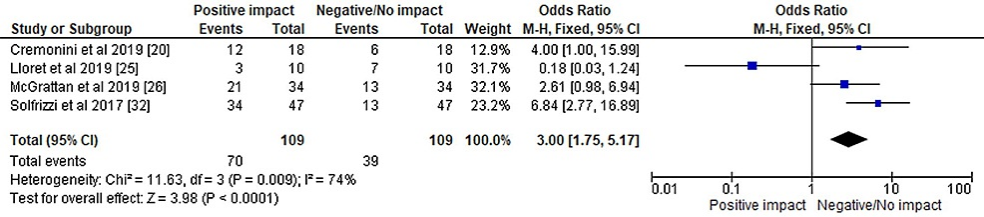
The odds ratio of selected systematic reviews represented on a forest plot after their meta-analysis where the positive impact of dietary interventions compared to the negative to negligible impact of dietary patterns was assessed

The results of the meta-analysis have been depicted in Figures 3-5, respectively.

Figure 3 is the forest plot that displays the results of a meta-analysis of selected randomized controlled trials assessing the impact of dietary interventions on AD. The odds ratio (OR) of the impact is reported as 0.20 with a 95% confidence interval (CI) of 0.15-0.26. The CI does not include 1, indicating a statistically significant impact of dietary interventions on AD. The heterogeneity of the included studies is reported as chi² = 5.45, df = 5 (P = 0.36), and I² = 8%, indicating low heterogeneity between the studies. The test for overall effect is reported as Z = 12.26 (P < 0.00001), indicating a strong overall effect of the dietary interventions (KD and MD or a combination of both) on AD. In summary, the forest plot shows a statistically significant OR of 0.20 with a narrow CI of 0.15-0.26, indicating a positive impact of dietary interventions on AD. The low heterogeneity (I² = 8%) and strong overall effect (Z = 12.26, P < 0.00001) support the validity of the meta-analysis results.

The forest plot in Figure 4 displays an OR of 0.59 with a 95% CI ranging from 0.53 to 0.67 of selected cross-sectional, observational, and cohort-based studies after their meta-analysis where the positive impact of dietary interventions compared to negative to negligible impact of dietary patterns was assessed. The heterogeneity among the studies was significant, with chi² = 19.47 and df = 5 (P = 0.002) and I² = 74%, indicating considerable variation between studies. The test for overall effect was statistically significant, with Z = 8.71 and P < 0.00001, suggesting that the observed effect of dietary interventions on the outcome of interest was not likely due to chance.

The forest plot represented in Figure 5 displays an OR of 3.00 with a 95% confidence interval (1.75, 5.17) of selected systematic reviews after their meta-analysis where the positive impact of dietary interventions compared to the negative to negligible impact of dietary patterns was assessed. The summary estimate indicates that the odds of the positive impact of dietary interventions were slightly higher compared to the negative to negligible impact of dietary patterns. Heterogeneity among studies is observed with chi² = 11.63, df = 3 (P = 0.009), and I² = 74%. This suggests that there is moderate to high heterogeneity among the included studies. The test for overall effect is Z = 3.98 (P < 0.0001), indicating that the overall effect of dietary interventions compared to dietary patterns is statistically significant (Table 1).

**Table 1:** Description and outcomes as observed in the studies selected for the systematic review CFU: colony-forming unit; g/day: grams per day.

Author and year of study	Sample size; mean age	Study design	Study description/intervention	Study outcome/inference
Agnihotri et al. (2020) [15]	-	Literature review	Through this literature review, the authors discussed epidemiological data that revealed exposure to environmental toxins, primarily pesticides, metals, and solvents, could increase the likelihood of developing neurodegenerative diseases. They also talked about how mitochondrial function affects cellular respiration, metabolism, energy production, intracellular signaling, and apoptosis.	The fundamental characteristics of Alzheimer's disease (AD) include protein misfolding and mitochondrial dysfunction, according to the authors. They also discovered that severe oxidative stress, which is a key factor in the etiology of neurodegeneration, could be brought on by the inhibition of antioxidant mechanisms in mitochondria. To ensure that the mitochondria function normally and to reduce the amount of oxidative damage, it was essential to maintain the primary antioxidant systems, primarily glutathione peroxidase and superoxide dismutase.
Berti et al. (2015) [16]	52 individuals; 54 years	Cross-sectional study	Dietary intake of 35 nutrients linked to cognitive performance and AD was evaluated. The entire nutritional panel was used to create nutrient patterns using principal component analysis.	The nutrient combination identified as being potentially protective against AD was characterized by a higher consumption of fresh fruits and vegetables, whole grains, fish, and low-fat dairy products. In contrast, this combination showed a lower intake of sweets, fried potatoes, high-fat dairy products, processed meat, and butter.
Berti et al. (2018) [17]	70 patients; 30-60 years	Cross-sectional study	70 cognitively normal participants between the ages of 30 and 60 years, who had clinical, neuropsychological, nutritional, and imaging biomarker tests at least two years apart were assessed in this study. 34 of them had better Mediterranean diet (MD) adherence (MD+), while 36 had lower MD adherence (MD). Volumes of interest and statistical parametric mapping were employed to compare AD biomarkers longitudinally and cross-sectionally between groups.	In terms of clinical and cognitive measurements, Mediterranean diet (MD) groups were comparable. The MD group initially displayed lower fluorodeoxyglucose-positron emission tomography (FDG-PET) cerebral metabolic rate for glucose (CMRglc) and increased Pittsburgh compound B-positron emission tomography (PiB-PET) deposition in AD-affected areas when compared to the MD+ group. In comparison to the MD+ group, the MD group displayed longer-lasting CMRglc decreases and Pittsburgh compound B increases in certain locations. On magnetic resonance imaging (MRI), no changes were seen. Increased MD adherence was thought to offer protection from AD for 1.5 to 3.5 years.
Chen et al. (2016) [18]	121 patients; >60 years	Randomized control trial	Patients with a recent AD diagnosis were divided into two groups at random and treated with folic acid supplementation for 6 months in either the intervention group or the control group.	The (amyloid beta) A𝛽 42/A𝛽 40 ratio was greater in the intervention group, indicating that folic acid was helpful for AD patients and that inflammation may have had a significant impact on the therapeutic role of folic acid in AD sufferers.
Chen et al. (2022) [19]	60 patients; 60-80 years	Observational study	This study aimed to investigate the link between oral-gut bacteria and markers of systemic inflammation.	In patients with AD, it was found that anti-inflammatory diets appeared to be associated with an increase in the abundance of helpful microbes in the oral-gut axis, whereas microbes associated with specific inflammatory markers and inflammation accumulation might have driven a shift from normal microbial composition to pathogenicity.
Cremonini et al. (2019) [20]	18 studies	Systematic review	This narrative review's objective was to provide a summary of the preclinical and clinical research that had been done on various types of dietary interventions.	The authors concluded that while there was promising new research with regard to the objectives of the study, there was still much work to be done before these dietary regimens could be applied to humans, and in particular patients with Alzheimer's disease.
Engelhart et al. (2002) [21]	5395 individuals; >55 years	Observational study	5395 patients in total who had a trustworthy dietary evaluation at baseline (1990–1993) and were at least 55 years old, dementia-free, not confined, and out of institutions. Participants were reexamined in 1993–1994 and 1997–1999, and incident dementia was continually tracked.	After an average follow-up of 6 years, 197 patients, 146 of whom had AD, developed dementia. Consumption of antioxidant supplements and high vitamin C and vitamin E intake were all associated with a lower risk of Alzheimer's disease when the baseline was adjusted for age and sex.
Feart et al. (2009) [22]	1410 individuals; ≥65 years	Prospective cohort study	This study involved 1410 adults from Bordeaux, France who were enrolled in the three-city cohort in 2001-2002 and followed up with at least twice over the course of five years. Four neuropsychological tests were used to measure cognitive performance.	It was observed that following MD was linked to lower levels of cognitive decline.
Freund et al. (2014) [23]	33 individuals	Randomized double-blind control trial	33 patients in all, 18 of whom received the n-3 fatty acid (FA) supplement, and 15 of whom received a placebo, were enrolled in the trial.	Eicosapentaenoic acid (EPA), docosahexaenoic acid (DHA), and total n-3 FA levels in the cerebrospinal fluid (CSF) (and plasma) significantly increased in the n-3 FA supplement group at 6 months, but not in the placebo group. Contrary to DHA, EPA and n-3 docosapentaenoic acid changes in CSF and plasma levels were closely associated.
Kincaid et al. (2021) [24]	-	Literature review	The authors of this literature review explained how adopting a balanced diet could reverse imbalances in the gut flora, benefiting the brain and lowering the risk of AD. The authors also shed light on gut-brain axis-based studies relating to food, gut microbiota, and AD pathogenesis.	The gut-brain axis was significantly influenced by bacteria living there, and they may have contributed to the pathophysiology of AD. Diet, one of the most potent regulators of gut microbiota, was found to have a significant impact on both AD pathogenesis and brain health. Short-chain fatty acids, pro-inflammatory substances, and neurotransmitters, among other gut microbiota metabolites, may have also influenced the etiology of AD.
Lloret et al. (2019) [25]	10 studies	Systematic review	Through this systematic review, the scientists examined research on Alzheimer's disease that had positive outcomes and those that had negative results. They also analyzed the reasons why vitamin E treatment can sometimes improve cognition but not always.	The authors came to the conclusion that clinical research had produced unreliable results regarding the impact of vitamin E on the likelihood of developing Alzheimer's disease. It was also not clear whether vitamin E levels were genetically linked to the risk of developing AD or whether supplementing with the vitamin was helpful in slowing the progression of dementia. Additionally, they discovered that there were more studies demonstrating a drop in plasma vitamin E levels in AD patients than studies demonstrating the opposite.
McGrattan et al. (2019) [26]	34 studies	Systematic review	The authors examined pertinent papers to address the potential neuroinflammatory effects of diet on cognitive function and evaluated the evidence for age-related cognition and healthy dietary patterns.	It was found that dietary factors may have affected cognitive aging through a number of inflammatory pathways. However, there was a shortage of information from human studies, and the precise mechanisms relating nutrition to cognitive performance remained opaque. To explore diet-associated neurological change from the earliest through the latter phases of cognitive decline, further dietary intervention trials were required. The authors further recommended that including neuroimaging measures in intervention studies would help researchers better understand how dietary changes affect neuroinflammation in the aging brain.
Nagpal et al. (2019) [27]	17 individuals; 64.6 years	Randomized double-blind control trial	Mediterranean-ketogenic diet (MMKD) versus American Heart Association diet (AHAD) intervention was compared in a randomized, double-blind, cross-over, single-center pilot trial with 17 participants, 11 of whom had mild cognitive impairment and 6 of whom had normal cognitive function. The subjects completed a 6-week MMKD and an AHAD intervention, with 6-week washout intervals in between.	According to the findings, certain gut microbial signatures might have indicated moderate cognitive impairment, and the MMKD may have the ability to alter the gut microbiome and metabolites in connection with improved CSF AD biomarkers, as assessed by measurements of the gut flora, fecal short-chain fatty acids (SCFAs), and AD indicators in CSF such as amyloid β (Aβ)-40 and Aß-42, total tau, and phosphorylated tau-181 (tau-p181) to quantify the cognitive changes.
Nagpal et al. (2020) [28]	17 individuals; 64.6 years	Randomized double-blind control trial	The authors measured the gut mycobiome by sequencing the fungal ribosomal RNA internal transcribed spacer 1 gene (rRNA ITS1) gene in 17 older adults (11 with mild cognitive impairment (MCI) and 6 with cognitively normal (CN)) before and after a 6-week intervention of MMKD and the American Heart Association Diet (AHAD), and they found a correlation between the gut bacteria and AD markers in CSF.	The study identified mycobiome signatures that are unique to MCI patients and showed how different diets can modify the mycobiome in MCI patients in conjunction with AD markers and networks that control the co-regulation of fungi and bacteria.
Omar (2019) [29]	-	Literature review	To slow the progression of AD, this review analyzed various dietary approaches that have been tried, such as caloric restriction (CR), dietary approaches to stop hypertension (DASH), ketogenic diets (KD), the Mediterranean diet (MedDi), and the Mediterranean DASH diet Intervention for neurological delay (MIND) diet.	Using olive biophenols as a possible preventative mechanism, this review supported the idea that the MD and MIND diets have the ability to sustain cognitive function as nonpharmacological agents against AD pathology. It also identified gaps and suggested future research approaches.
Romanenko et al. (2021) [30]	-	Literature review	The potential causes of AD, as well as the potential of probiotics and prebiotics in therapeutic modulation of contributed pathways, were also discussed in this review. The research findings that supported the role of intestinal microbiota in the connection between nutritional factors and the risk for Alzheimer's disease onset and progression were also summarized.	According to the authors' assessment of the available data, food and lifestyle changes may have had an impact on cognitive performance. In keeping neural homeostasis and delaying cognitive decline, several foods appeared to be helpful. Along with it, the strongest supporting data for whole-diet strategies including MD, DASH, and MIND were revealed. The makeup and functional activity of the gut microbiome play a critical role in how nutrition affects cognitive function and the risk of AD.
Rusek et al. (2019) [31]	-	Literature review	This review sought to investigate the impact of the ketogenic diet (KD) in the course of Alzheimer's disease and to highlight particular elements of the nutritional profile that would support the use of dietary interventions as a treatment for the condition.	Based on the few clinical trials and animal studies, it was shown that KD had positive impacts on improving cellular metabolism and mitochondrial function. In older people with AD, it was linked to increased cognitive performance. The degree and length of ketosis were factors in how the cognitive outcomes improved. However, the authors cautioned for further studies to establish the credibility of this dietary pattern.
Solfrizzi et al. (2017) [32]	47 studies	Systematic review	In this study, the authors systematically reviewed observational studies on dietary factors and late-life cognitive disorders that had been published in the three years prior (2014–2016). These studies examined dietary patterns, foods and food groups, dietary micro- and macronutrients, and potential underlying mechanisms of the proposed associations.	The National Institute on Aging-Association Alzheimer's guidelines for Alzheimer's disease (AD) and cognitive decline caused by AD pathology provided some data from the reviewed literature suggesting a clear connection between nutrition and changes in the structure and activity of the brain. Additionally, there was mounting evidence that certain combinations of foods and nutrients may function synergistically to impart health benefits that are stronger than those offered by their individual dietary components. Increased adherence to a Mediterranean-style diet in particular was linked to a slower rate of cognitive impairment.
Tamtaji et al. (2019) [33]	79 patients; 55-100 years	Randomized, double-blind control trial	The participants in this randomized, double-blind, controlled clinical trial consisted of 79 AD patients. For a period of 12 weeks, patients were randomized to receive selenium (200 g/day) together with a probiotic containing *Lactobacillus acidophilus*, *Bifidobacterium bifidum*, and *Bifidobacterium longum* (2 109 CFU/day each), selenium (200 g/day) or a placebo.	Overall, it was discovered that probiotics and selenium co-supplementation to patients with AD for 12 weeks improved metabolic profiles and cognitive function compared to placebo.
Taylor et al. (2018) [34]	15 participants; 73.1 years	Observational study	In participants with Alzheimer's disease, the authors evaluated the viability and cognitive effects of a ketogenic diet (KD). In the trial, patients with clinical dementia rating (CDR) 0.5, 1, and 2 had a 3-month KD with medium-chain triglyceride supplementation, followed by a 1-month washout. We collected data on safety, food history, serum b-hydroxybutyrate, and urine acetoacetate.	The findings suggest that the ketogenic diet could potentially be a viable strategy for some patients with AD, as it was able to induce ketosis in the majority of participants. However, the diet also presented including caregiver burden and adverse effects related to medium chain triglycerides.
Tomaszewski et al. (2020) [35]	275 patients;	Randomized control trial	Through this work, the authors aimed to shed further light on the impact of (apolipoprotein E) APOE ε4/ε4 on hippocampus sizes following DHA supplementation as well as the ratio of plasma DHA and EPA to arachidonic acid (AA). Therefore, plasma fatty acids and APOE genotype were measured in 275 volunteers who were randomly assigned to receive DHA supplements for 18 months or a placebo. A subgroup of these subjects had lumbar punctures (n = 53) and brain MRI imaging (n = 86).	When compared to APOE 4/4 carriers, plasma DHA/AA levels considerably increased in APOE ε3/ε3 and APOE ε2/ε3 carriers after receiving DHA treatment. Comparing APOE ε2/ε3 carriers to ε4/ε4 carriers, ε2/ε3 carriers experienced higher increases in plasma EPA/AA and less loss in the left and right hippocampus volumes. Strong correlations were found between the changes in plasma and cerebrospinal fluid DHA/AA. Only in APOE4 non-carriers, there was a reduced decline in the right hippocampus volume associated with a higher baseline and increase in plasma EPA/AA.

Discussion

As per the statistical findings, in certain cases, dietary interventions appeared to provide a protective effect, reducing the risk of developing AD. This means that changes in diet, such as incorporating certain foods or nutrients, can help delay or potentially prevent the onset of this condition. However, the effectiveness of these interventions varied across the studies. Some research indicated a strong protective effect, while others showed a less pronounced but still beneficial impact. Interestingly, in other cases, dietary interventions did not appear to be more beneficial than maintaining regular dietary patterns. This suggests that the effectiveness of dietary changes may depend on various factors, such as the specifics of the intervention, the population studied, and perhaps individual genetic differences.

The multifaceted nature of the dietary regimens found in our chosen studies lends them strength. Contrary to popular belief, nutrient-rich foods can interact with one another and possibly have synergistic effects on different cellular and metabolic signaling pathways. However, in vivo studies involving human subjects present ethical considerations, particularly when the research involves interventions that could impact the health of participants. As a result, such studies must undergo rigorous ethical review to ensure that they comply with the necessary ethical standards. Moreover, in vitro and in vivo studies investigating the effects of dietary patterns on the brain often involve complex interventions, which may include the consumption of multiple nutrients and food components. Isolating the effects of specific dietary patterns from the confounding effects of other dietary components and lifestyle factors can be challenging [36].

The precise mechanisms underlying the neuroprotective benefits connected with the MD and the other dietary patterns are still not fully understood, despite the fact that it is generally acknowledged that these dietary patterns play a role in combating a number of biological processes implicated in the pathogenesis of AD, such as oxidative stress, neuroinflammation, neurovascular dysfunctions and hypoperfusion, disruption of the gut-brain axis, and impairment of hippocampal neurogenesis. It is also likely that MD can alter specific metabolic pathways, even though the majority of the data for these pathways originated from animal research and has to be further assessed and confirmed [37,38]. By reducing cardiovascular risk factors like cholesterol levels, blood glucose, and blood pressure, MD has been shown to indirectly improve cognition on a systemic level [37,38].

Microglial cells give the brain its first and most important immune defense and are a well-established feature of neuroinflammation and one of the important factors in AD etiology [39-42]. The accumulation of A-peptides contributes to the development of AD by causing abnormal free radical production, the release of pro-inflammatory cytokines and chemokines, and the degradation of neuroprotective compounds like retinoids that support adult neurogenesis in the hippocampal formation [43-46]. The disease gets worse and becomes more severe as a result of this persistent inflammatory response, which also increases the amount of A plaques generated and deposited and ultimately results in neuronal death [42,43]. Numerous exogenous or endogenous factors, including genetics [47-52], might exacerbate the innate immune response.

Resveratrol is another anti-inflammatory compound that is hypothesized to assist in combating the pathogenesis of AD [48]. The generation of tau and A plaques is decreased [49,50], pro-oxidative stress proteins are downregulated [51,52] and neuroinflammation is decreased [53-58]. Resveratrol has also been associated with a variety of other functions [59,60]. A diet high in polyunsaturated fatty acids (PUFA) modifies the fibrillar/prefibrillar A oligomer ratio to reduce the density of hippocampal A plaques (the former is less toxic), thereby producing mild behavioral improvements in a transgenic rodent model of AD [61-64]. The fluidity of membranes and the activity of enzymes in neurons may be impacted by dietary intake of omega-3 and omega-6 PUFA and monounsaturated fatty acids (MUFA), which may then modify the architecture and functions of the brain [65,66].

A plant-based diet rich in fiber, vitamins, and polyphenols has shown promising benefits for memory and brain health, according to current evidence [67-69]. One study proposed [67] dietary and lifestyle guidelines for AD prevention, emphasizing the importance of plant-based diets with abundant fruits, vegetables, whole grains, legumes, and nuts. They also recommended reducing saturated and trans fats while increasing the intake of vitamins E and C, folate, and antioxidants. In another study on the Mediterranean-DASH (Dietary Approaches to Stop Hypertension) diet intervention for neurodegenerative delay (MIND) diet [36], which combines elements of the Mediterranean and DASH diets, adherence to the plant-based MIND diet was associated with a decreased risk of developing AD. The World Health Organization (WHO) published guidelines in 2019 [68] that recommended a healthy diet, such as the Mediterranean-style diet, which focuses on fruits, vegetables, legumes, and whole grains, to reduce the risk of cognitive decline and dementia. Furthermore, Roman et al. [69] reviewed the Mediterranean diet's role in preventing stroke, age-related cognitive decline, and AD. Their analysis highlighted the potential benefits of polyphenols in fruits, vegetables, cereals, coffee, tea, cacao, and wine, as well as long-chain omega-3 fatty acids in fish, probiotics, and vitamins for brain health. Collectively, these studies provide evidence supporting the positive impact of a plant-based diet containing fiber, vitamins, and polyphenols on memory and brain health. However, it is important to acknowledge that dietary supplements may not provide significant benefits in preventing cognitive decline or AD when individuals do not have underlying deficiencies. A study conducted by Roman et al. [69] delves into the potential preventive effects of various dietary components, including long-chain omega-3 fatty acids found in fish, polyphenols present in fruits, vegetables, cereals, coffee, tea, cacao, and wine, as well as probiotics and vitamins. Through their analysis, the researchers discuss the role of these dietary factors in preventing stroke, age-related cognitive decline, and AD. Importantly, their conclusions highlight that the preventive effects observed in the context of these dietary components are most pronounced when there are deficiencies or imbalances present.

Speaking about the limitations of our systematic review, there is significant heterogeneity in the study characteristics as evident from our findings mentioned in Table 1. The variations in the mean age of the study participants as well as differences in dietary patterns across different geographical areas also compound the heterogeneity of the study. Hence, we believe that more studies assessing the impact of various dietary patterns on the well-being of patients of AD are crucial so that a concrete, targeted nutritional intervention can be designed that has lesser levels of ambiguity than what was observed in the studies selected in our investigation.

## Conclusions

Our selected studies and subsequent meta-analyses have suggested that dietary interventions and nutritional corrections may play a role in delaying the onset of AD and the debilitating levels of dementia associated with it. However, the observations are not entirely unambiguous, as a few of the selected studies did not report any significant changes in the patients' condition. Consequently, we believe that the relationship between nutritional interventions and AD requires further research and more extensive metadata to be definitively proven. Given the breadth of the scientific literature reviewed, it is difficult to identify a single "best" diet for reducing or controlling AD. This challenge arises from the diversity of study designs, interventions, and outcome measures. However, the reviewed studies consistently suggest that certain dietary factors may have a protective effect against AD. These factors include adherence to a Mediterranean diet, consumption of fresh fruits and vegetables, whole grains, fish, and low-fat dairy products, as well as the incorporation of antioxidant-rich foods into the diet. Moreover, specific interventions, such as supplementation with folic acid or n-3 fatty acids, and following certain dietary patterns like the MD, have shown promising results in terms of potential improvements. Nonetheless, more research is needed to confirm these findings and to develop comprehensive dietary guidelines for managing AD.
